# Evaluation of different Mediterranean essential oils as prophylactic agents in anisakidosis

**DOI:** 10.1080/13880209.2016.1247880

**Published:** 2016-12-09

**Authors:** Magdalena Gómez-Mateos Pérez, Concepción Navarro Moll, Gema Merino Espinosa, Adela Valero López

**Affiliations:** Department of Parasitology, Faculty of Pharmacy, University of Granada, Granada, Spain

**Keywords:** *Anisakis* larvae, components of essential oils, prophylactic action, biocidal activity, Mediterranean plants

## Abstract

**Context:***Anisakis* Dujardin 1845 (Anisakidae) nematodes can cause gastrointestinal and allergic diseases when humans eat raw or undercooked seafood containing larvae. There is currently no drug available in the market against this parasitic disease, and the study of plant-derived molecules could be useful in the discovery of effective compounds.

**Objective:** This research assesses the biocidal activity of a range of essential oils (EOs) from some Mediterranean plants against larvae found in the musculature of fresh fish.

**Materials and methods:** EOs composition was analyzed by gas chromatography-mass spectroscopy. All the EOs were diluted at 5% v/v in olive oil to cover the fish with the solutions for 24 h. The larvae that abandoned the muscle and the larvae obtained from the artificial digestion of the fish were collected. Controls were carried out in parallel. Furthermore, Wistar rats were infected with the live larvae collected from the *in vitro* trials in order to find any larvae that may have penetrated the gastrointestinal wall.

**Results:** Between 60.8% and 87.6% of parasites treated with EOs abandoned the fish muscle, and the highest *in vitro* mortality rate was achieved with oregano EO (53.9%). Rats previously treated with oregano, cumin and Spanish lavender EOs showed no detectable lesions in the digestive tract due to the infection with larvae.

**Conclusions:** Oregano (*Origanum vulgare* L. Lamiaceae), cumin (*Cuminum cyminum* L. Apiaceae) and Spanish lavender (*Lavender stoechas* L. Lamiaceae) EOs could be used as promising ingredients in the development of products for the control of anisakiasis.

## Introduction

The consumption of marine fish has increased over recent decades, raising the number of cases of human anisakidosis. The main agent responsible for this parasitosis is the third larval stage (L3) of *Anisakis* Dujardin, 1845 (Anisakidae), nematodes which are widely found in fish and cephalopods, with high mean prevalence and intensity rates in many species of commercial interest (Chía et al. 2010; Pekmezci 2014; Klapper et al. 2015). Human beings get infected when they consume raw or undercooked fish or cephalopods. According to various authors, these nematodes are highly resistant to the conditions created by traditional marinating methods; they are able to survive for periods of a few days up to several weeks, depending on the concentration of salt, acetic acid and marinating times (Karl et al. 1995; Sánchez-Monsalvez et al. 2005; AESAN 2007; Giarratana et al. 2012; Anastasio et al. 2015). All these circumstances lead to frequent reports of anisakidosis all over the world, with Japan at the top of the list with more than 2000 cases a year, due to high raw fish consumption in this country. When larvae invade the human gastrointestinal tract, they are usually found in the gastric cavity, they can also be located in various different areas of the intestine. Clinical symptoms range from digestive problems, which vary depending on the location of the larvae, to allergic reactions of varying severity, and sometimes both at the same time. In terms of prevention, various different prophylactic measures were proposed shortly after the first cases were diagnosed in humans. The European Union and WHO, now aware of the problem, stress the importance of freezing fish and seafood products destined for salting, marinating and cold smoking, at a temperature of −20 °C or lower for at least 24 h, or heating the fish to over 60 °C. For its part, the FDA advises freezing the fish at −20 °C for seven days or at −35 °C for 15 h in order to kill any larvae. Nevertheless, it would be of interest to have other alternative measures at our disposal in daily practices that guarantee the destruction of the larvae. To fulfil this aim, various studies reporting on the larvicidal effect of different natural products have been published, especially the essential oils and/or their components (Hierro et al. 2004, 2006; Navarro et al. 2008; Romero et al. 2012, 2014; Giarratana et al. 2014, 2015a,b; Gómez-Rincón et al. 2014; Valero et al. 2015). In this study, various essential oils (EOs) extracted from plant species present in the Mediterranean region have been evaluated. The specific purpose of this research was to study the potential action of these EOs on *Anisakis* larvae in the musculature of fish and later on to assess their infectivity on laboratory animals.

## Materials and methods

### Biological material

The host used in the different trials was blue whiting (*Micromesistius poutassou*) from the north of Spain, a fish with a high prevalence of parasitization by *Anisakis* L3 larvae, as shown by various authors (Chía et al. 2010).

### Natural products tested

The following EOs were tested against the *Anisakis* L3 larvae found in the muscle of the blue whiting: Spanish lavender (*Lavandula stoechas* L. Lamiaceae), cumin (*Cuminum cyminum* L. Apiaceae), lavender (*Lavandula spica* L. Lamiaceae), marjoram (*Origanum majorana* L. Lamiaceae), oregano (*Origanum vulgare* L. Lamiaceae), rosemary (*Rosmarinus officinalis* L. Lamiaceae) and thyme (*Thymus vulgaris* L. Lamiaceae). These products were provided by Sensient Fragrances (Granada, Spain). The composition of the EOs was analyzed by gas chromatography–mass spectroscopy (GC–MS) on an Agilent 7890A System (Santa Clara, CA) coupled with a Waters Quattro Micro GC mass spectrometer (Cerdanyola del Vallès, Spain). The column used was a phenyl dimethylpolysiloxane capillary column ZB-5MS (30 m × 0.25 μm thickness), supplied by Phenomenex (Torrance, CA), with helium as the carrier gas (flow rate = 1 mL/min). The samples were injected using the split mode (split ratio 1:1000), with injector temperature at 200 °C. Oven temperature was programed from 50 °C (2.5 min) to 200 °C at a rate of 4 °C/min, then kept at 200 °C for 8 min. Mass spectra were acquired in electron impact mode (70 eV). Components were identified by mass spectral fragmentation, by comparing Kovats-calculated (Dabrio 1973) retention indices with those of known constituents, and by using a mass spectral matching library search system (Adams 2007).

### In vitro *study*

All the EOs were diluted at 5% v/v in olive oil. The presence of parasitic larvae in the muscle of the hosts was confirmed by direct observation. About 7–9 fish were used for each EO, which were gutted, washed and placed in glass containers covered with each essential oil solution. The containers were closed and kept for 24 h at 4 °C. The larvae that had abandoned the muscle were then collected, and the fish was then washed and immediately submitted to artificial digestion in a pepsin-HCl solution (pH 2–2.4) for 45 min at 36 °C. Following this, the larvae were counted, both those found loose in the container and those obtained from the digestion of the muscle. Controls were carried out in parallel, submerging the fish in olive oil (carrier), in order to verify that it has no effect on the larvae. The viability of the parasites was determined in saline solution 0.9% at 24 h under a stereoscopic microscope, applying the following criteria of mobility: dead if no movement or response to stimulus were observed, apparently alive if there was movement when stimulated with a brush and alive if there was spontaneous movement.

### In vivo *study*

Forty-four female Wistar rats (weighing 140–150 g) were infected via a gastric probe with 4–8 active larvae mixed with 0.5 mL of water. The trials were conducted according to the following protocol: (a) many animals were infected with the live larvae that had abandoned the muscle during the immersion period in each of the EOs, (b) another lot consisted of animals infected with the larvae recovered from the fish after treatment and subsequent digestion. In order to establish the potential influence of the carrier oil on the viability of the larvae, a control lot was designed consisting of rats infected with the larvae obtained following the digestion of the fish submerged only in olive oil. Four hours later, the animals were sacrificed and immediately their organs were examined in detail to find any larvae that may have penetrated the wall of the gastrointestinal tract. Lastly, the digestive system was removed and opened under a stereoscopic microscope, recording the location of the larvae and the number of parasites and lesions found.

### Ethics statement

This study was conducted according to the principles specified in the Declaration of Helsinki, and under the Directive 2010/63/EU of the European Parliament and of the Council of the European Union (22 September 2010) and the Spanish Legislation (RD 53/2013).

## Results

### Chromatographic analysis

Table 1 shows the main components of the oils tested.

**Table 1.** Qualitative and quantitative composition of the essential oils assayed against *Anisakis* larvae performed by GC-MS.

Compound (%)	Spanish lavender	Cumin	Lavender	Marjoram	Oregano	Rosemary	Thyme
α-Thujene					0.49		0.02
α-Pinene	1.06	0.28	0.53	1.51	0.30	13.47	0.05
Camphene	1.30		0.24	0.37		5.86	0.06
Sabinene				1.54			
β-Pinene		7.30	0.55	2.38		2.39	
β-Myrcene		0.33		0.77	0.63	1.58	0.17
α-Terpinene					0.47		0.13
*p*-Cymene		12.25	0.18	0.49	3.32	2.15	17.74
Limonene	0.75		0.18	1.83		3.06	0.04
1,8-Cineole	5.17		19.58	68.05		27.98	0.14
Ocimene				0.48			
Δ4-Carene		10.47			1.30		0.41
Linalilo oxide			0.28	0.37	0.23		0.10
Fenchone	48.68		0.09				
Linanool			52.59	9.53	0.91	1.20	1.39
Camphor	39.98		17.33	0.73		23.32	
Lavandulol			1.79				
Santolina alcohol				1.25			
Borneol			1.71	0.76	0.09	4.97	0.47
Terpinen-4-ol		0.39	0.35	0.84	0.46	1.11	
α-Terpineol		0.82		4.83			
*o*-Cresol		0.24					
Isobornil format							0.05
Cuminaldehyde		34.11					
Linalilo anthranilate				2.29			
Felledral		0.72					
Bornyl formate	1.89						
Δ2-Caren-10-al		20.78				1.64	0.04
Δ3-C10-al		11.80					
Thymol					0.22		76.31
Carvacrol					88.39		2.41
Terpenyl acetate				1.00			
Aromadendrene				1.01	2.42	3.26	0.29

### Morphological identification

Examination under the microscope revealed that the 784 *Anisakis* L3 larvae obtained from the muscle of *M. poutassou* belonged to Type I.

### In vitro *studies*

**Figure 1.**
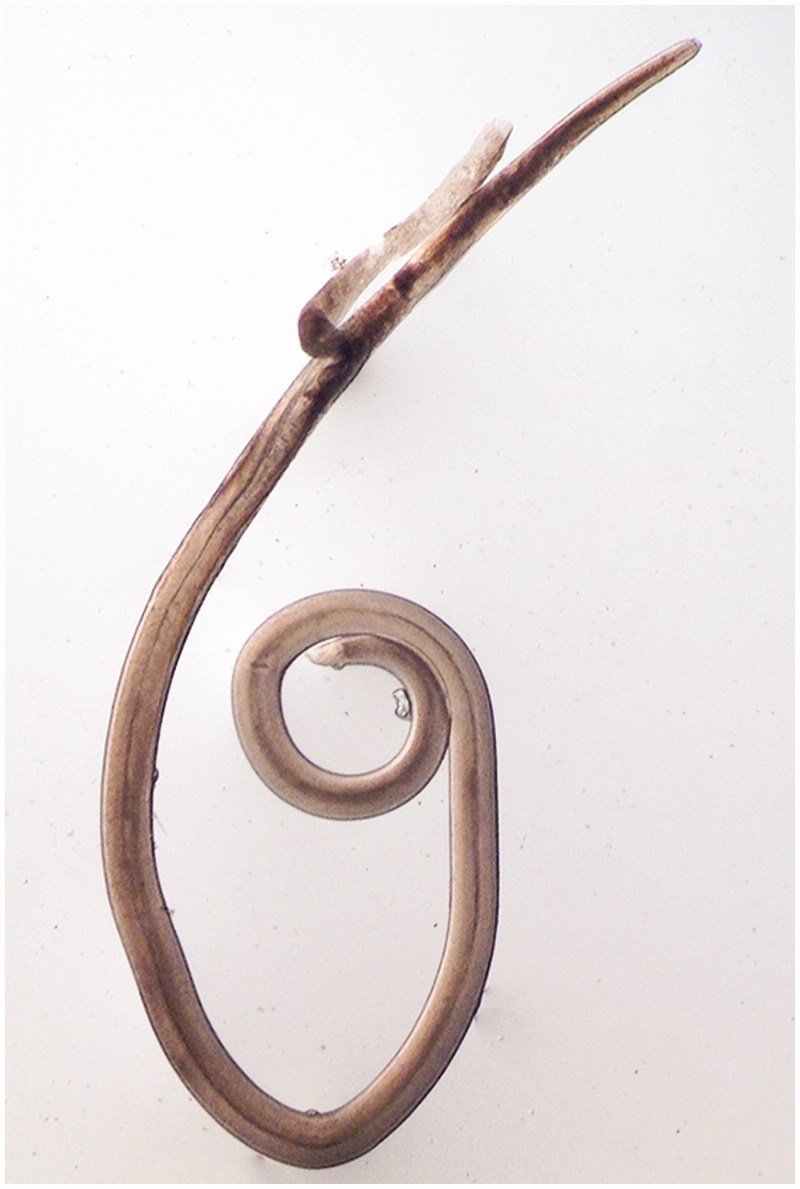
Larva L3 of *Anisakis* after being treated with essential oil of oregano *in vitro.* It can be observed in oesophageal and intestinal damage.

In the trials conducted with the parasitized fish treated with the Eos, a displacement of the larvae was generally observed from the parasitized tissues to the container in which the test was conducted. This migration was above 60% in all cases. Moreover, all the EOs tested produced a drop in the survival rate of the larvae. The highest total mortality rate was detected for oregano EO (53.9%), with the larvae showing severe oesophageal and intestinal deterioration (Figure 1). Cumin, thyme and Spanish lavender EOs proved less lethal than the oregano one (20.3, 11.4 and 3.5% mortality rates, respectively). For the lavender, marjoram and rosemary EOs the survival rate was 100%. For the larvae that remained alive, a reduction in spontaneous movement was detected, with the maximum reduction (65.8%) corresponding to larvae treated with cumin EO and the minimum (12.3%) to Spanish lavender EO; results in between these values were obtained for thyme (53.4%), lavender (31.7%) and oregano (31.4%) EOs. None of the larvae treated with marjoram and rosemary EOs required stimulation to be actively mobile. In the controls, all the larvae displayed great mobility. Table 2 displays data related to the larvae that abandoned the fish muscle or remained in it, and their survival rates and mobility following exposure to the different EOs.

**Table 2.** *In vitro* study of the effect of the EOs against the L3 of *Anisakis* type I.

Total L3		Container				Muscle			
		Total	NS	S	X	Total	NS	S	X
Spanish lavender	57	41 (71.9%)	32 (78.1%)	7 (17.1%)	2 (4.9%)	16 (28.1%)	16 (100%)	–	–
Cumin	237	196 (82.7%)	28 (14.3%)	123 (62.8%)	45 (23.0%)	41 (17.3%)	5 (12.2%)	33 (80.5%)	3 (7.3%)
Lavender	126	107 (84.9%)	71 (66.4%)	36 (33.7%)	–	19 (15.1%)	15 (79.0%)	4 (21.1%)	–
Marjoram	89	78 (87.6%)	78 (100%)	–	–	11 (12.4%)	10 (100%)	–	–
Oregano	102	62 (60.8%)	–	9 (14.5%)	53 (85.5%)	40 (39.2%)	15 (37.5%)	23 (57.5%)	2 (5%)
Rosmary	49	34 (69.4%)	33 (100%)	–	–	15 (30.6%)	15 (100%)	–	–
Thyme	88	69 (78.4%)	21 (30.4%)	38 (55.1%)	10 (14.5%)	19 (21.6%)	10 (52.6%)	9 (47.4%)	–

NS: L3 alive with no stimulus; S: L3 alive with stimulus; X = L3 dead.

### In vivo *studies*

This study was influenced by the *in vitro* results in terms of the number of larvae found alive and active following exposure of the fish to the different EOs. A total of 44 animals were infected with 218 larvae obtained in the *in vitro* trials, but the parasites only penetrated the digestive tract wall in five cases (11.4%). One rat had a lesion consisting of a tunnel measuring 2 × 1.5 mm with slight bleeding, located in the greater curvature of the stomach, and the larva was found partially embedded at the end of the tunnel. This lesion was caused by one of the L3 exposed to lavender EO that was collected from the container after abandoning the muscle.

The larvae treated with rosemary EO caused a similar tunnel-shaped lesion, although of greater length (4 × 1 mm), and a small ulcer (1.5 × 1 mm) surrounded by a bleeding halo; the larvae, as in the previous case, were found embedded in the greater curvature of the stomach. A third ulcer was also observed in the small intestine accompanied by signs of bleeding. The first lesion was caused by one of the L3 that had abandoned the fish muscle; the other two were caused by L3 that had remained in the muscle. Furthermore, L3s treated with rosemary EO and taken from the muscle produced two small ulcers (1 × 1 mm) in another infected rat; in one of these ulcers the parasite was found stuck in the gastric wall of the greater curvature, showing no visible signs of bleeding, and in the other it was located in a reddish area of the small intestine.

In one of the animals infected with larvae exposed to thyme EO and collected from the container, a lesion of 2 × 1 mm was found in the gastric fundus. In another rat, one larva was found in the body cavity with no visible lesions; this larva had been treated with marjoram EO and had abandoned the fish muscle during the process.

All the parasites recovered from the rats that had been taken from the fish muscle treated with EOs were found alive, except for the 22.9%, which had been in contact with cumin EO. The greatest number of larvae were recovered from the small intestine (49.1%), followed by the stomach (41.3%) and only 6.0% from the cecum. Table 3 shows the number of rodents infected and larvae inoculated, the lesions produced and their locations. With regard to the control lot, made up of animals that were infected with larvae from untreated fish muscle subjected to digestion, lesions were observed in all cases. These consisted of tunnels and ulcers similar to those described above and were found mainly in the gastric body (72.7%) and the remaining 27.3% in the small intestine (Table 3).

**Table 3.** Sinopsis of the *in vivo* trials.

Product	Origin of L3	No of rodents	No of L3 inoculated	Lesions (number)/Location
Control	Muscle	6	36	11/8 Stomach and 3 small intestine
Spanish lavender	Container	3	16	–
	Muscle	2	10	–
Cumin	Container	7	28	–
	Muscle	2	5	–
Lavender	Container	4	25	1/Stomach
	Muscle	3	15	–
Marjoram	Container	6	30	–
	Muscle	2	10	1/Stomach
Oregano	Container	–	–	–
	Muscle	3	15	–
Rosmary	Container	4	20	2/Stomach
	Muscle	3	14	2/Stomach and small intestine
Thyme	Container	3	17	1/Stomach
	Muscle	2	10	–

## Discussion

Human anisakidosis presents a risk to public health that must be reduced through basic prevention. Steps to control the disease include various measures and checks to detect and kill the larvae in fish, thus preventing infection of the consumer. The main recommendations are freezing the fish or exposing it to temperatures over 60 °C. However, essential oils have long been recognized as effective plant-based antimicrobials and have been used to preserve foods for centuries, thus they could be good alternatives to the abovementioned methods to protect against anisakidosis. Indeed, all seven essential oils tested in this study did somehow appear to stimulate a high percentage of the *Anisakis* L3 in the fish musculature to abandon it and migrate into the container (Table 2): the values were ≥70% for the lavender, Spanish lavender, marjoram, cumin, thyme and rosemary EOs, and slightly lower for the oregano one (60.8%). These data are of particular interest given that the muscle is the part of the fish that humans consume, and thus the reduced presence of parasites in this part would reduce the risk of contracting this disease. Furthermore, during this process a certain number of larvae in the fish died or lost spontaneous mobility on coming into contact with the essential oils (Table 2): the two most effective EOs in this sense were cumin and oregano, for which only 13.9% and 14.7% of the larvae, respectively, exhibited spontaneous movement. The favourable results obtained for these two EOs against *Anisakis* larvae are in keeping with those described by Valero et al. (2006), although this study was conducted under different conditions. The GC–MS analysis of cumin EO revealed the presence of its major components: cuminaldehyde (34.1%), 2-caren-10-al (20.8%), *p*-cymene (12.3%) and 3-caren-10-al (11.8%); carvacrol was detected in a high proportion (88.4%) in the oregano oil, while a low proportion of *p*-cymene (3.3%) and aromadendrene (2.4%) was found (Table 1). These compounds have been identified in both EOs, displaying inhibitory activity against certain pathogenic agents (Martinez-Velazquez et al. 2011; Dussault et al. 2014; Kedia et al. 2014; Fournomiti et al. 2015). The chemical profile of essential oil of a particular plant species shows different chemotypic variations because of ecological and geographical conditions, age of plant and time of harvesting (Prakash et al. 2010). Such chemotypic variations would definitely affect the biological activity of the essential oil (Kedia et al. 2014). Thyme EO exhibited less anti-anisakis activity: it only achieved the death of 11.4% of larvae but a high percentage of larvae (53.4%) needed stimulus to move. For thyme EO at the same concentration (5%), Giarratana et al. (2014) indicated 100% mortality after 14 h of treatment; this difference in larvicidal activity could be due to the test conditions and the chemical composition of the essential oil used by these authors [thymol (50%), linalool (7%), carvacrol (3%), α-pinene and β-pinene (6%)] in comparison with the thyme EO employed by us [thymol (76.3%), *p*-cymene (17.7%), carvacrol (2.4%) and linalool (1.4%)] (Table 1). The phenolic molecules thymol and carvacrol have been observed as the more active constituents (di Pasqua et al. 2005), being principally responsible for the antimicrobial activity, for example, against *E. coli*, *K. oxytoca* and *K. pneumoniae* (Fournomiti et al. 2015). The rest of the EOs did not display biocidal activity against the L3s.

With regard to the *in vivo* trials, the action of oregano, cumin and Spanish lavender EOs on the L3s in the fish muscle resulted in the larvae not causing any lesions in the animals’ digestive tract. The antimicrobial activity of essential oils, which are markedly lipophilic compounds, is related to their chemical composition, in which there is a predominance of terpenic derivatives (mono- and sesquiterpenes). Although its mechanism of action has not yet been fully explained, it is likely that in most cases it involves disruptions of the membrane, increasing the permeability of the bacterial cytoplasmic membranes, and of the mitochondrial membranes in eukaryotic organisms (Cowan 1999; Burt 2004). A decrease in the hydrophobicity of certain terpenes by the addition of methyl groups reduces their antimicrobial activity (Mendoza et al. 1997; Usano 2012). Furthermore, the main component of oregano EO, carvacrol, is known to interact with the cell membrane of some organisms, where it dissolves in the phospholipid bilayer and aligns between the fatty acid chains. The distortion of the physical structure causes expansion of the membrane, thus bringing about an increase in its permeability (García-García & Palou-García 2008). Moreover, carvacrol and 1,8-cineole, alone or in combination, severely affected the viability of the bacteria and caused dramatic changes in the cell membrane permeability, leading to cell death, as observed by confocal laser microscopy (de Sousa et al. 2015).

In the case of cumin EO, the activity of its main component, cuminaldehyde, against various pathogenic agents including *Anisakis* L3 larvae has been described, although the exact way in which it acts is still unknown (Hierro et al. 2004; Yeom et al. 2012).

With regard to the action of Spanish lavender EO, we should point out that although it did not show larvicidal action in the *in vitro* studies, in the *in vivo* trials no lesions were observed in the infected animals treated with this EO. The chemical composition identified in this oil was: fenchone (48.7%), camphor (40.0%) and 1,8-cineole (5.2%), so the EO analyzed could be considered a camphor-fenchone chemotype, according to Granger et al. (1973). Others authors detected these molecules in the Spanish lavender EO as its most representative components (Zrira & Benjilal 2003; Dadalioglu & Evrendilek 2004), although differences in the percentages have been found: fenchone, 14.9–75.5%; camphor, 2.5–56.2%; and 1,8-cineole, 0.2–8% (Ristocelli et al. 1998). Considering the high amount of fenchone detected in our oil, the difference in activity between the *in vitro* and *in vivo* trials for this EO may be related to a possible conversion, facilitated by the gastric pH, of fenchone into its alcohol derivative, fenchol, a compound which has been shown to be effective against G + and G − bacteria, as well as against various fungi (Kotan et al. 2007; Guleria et al. 2013).

It should also be highlighted that in the case of the larvae treated with marjoram EO, although one larva was found in the body cavity of the animal, there were no visible signs of lesions in the digestive tract. This leads us to speculate that this EO, whose main component is 1,8-cineole (68.1%), may have favoured the reabsorption of the haemorrhage. This could be due to the anti-inflammatory activity of 1,8-cineole which, in agreement with the results obtained by Abu-Darwish et al. (2013) would exert an anti-inflammatory effect through a mechanism involving the inhibition of LPS-induced NO production. This molecule has also been shown to be the majority constituent in the chromatogram of this EO, followed by terpenoids (Waller et al. 2016).

## Conclusions

The essential oils tested in this work provoked the migration of the *Anisakis* larvae out of the fish, therefore they might well be added to the marinated fish plates. This fact, together with the biocidal action of some of them, particularly Spanish lavender, cumin and oregano EOs, are promising active ingredients that could be used in the development of effective products for the control of anisakidosis. Further studies on safety and palatability of the marinating with essential oils should be carried out.
